# 

**Published:** 1963-07

**Authors:** 


BRISTOL MEDICO-CHIRURGICAL JOURNAL

*(Incorporating the Medical Journal of the South-West)*

Established 1883

VOL. 78 (iii) July 1963 NO. 289

### Contents

| | | |
|---|---|---|
| THE DOCTOR IN THE LAW COURTS | *Lord Justice Donovan* | 65 |
| HOW TO WIN A "HOUSE JOB" | *A. J. G. Dickens* | 75 |
| CATARACT | *P. Jardine* | 84 |
| PAROXYSMAL NOCTURNAL HAEMOGLOBINURIA | *Clinical-Pathological Conference* | 92 |
| OBITUARY | | 102 |
| REPORTS FROM SOCIETIES | | 104 |
| NOTES AND NEWS | | 105 |
| EXAMINATION RESULTS | | 105 |
| APPOINTMENTS | | 105 |

*PUBLISHED QUARTERLY*

*ANNUAL SUBSCRIPTION* **16/-** *POST FREE*

PUBLISHED UNDER THE AUSPICES OF THE
BRISTOL MEDICO-CHIRURGICAL SOCIETY

### *Editorial Committee*

Dr. N. J. Brown, Southmead Hospital, Bristol. *Joint Editor.*

Dr. A. B. Raper, Department of Pathology, Bristol Royal Infirmary. *Joint Editor.*

Dr. J. Macrae, Ham Green Hospital.

Dr. R. C. Wofinden, Central Clinic, Bristol.

Mr. A. E. S. Roberts, Medical Library, University of Bristol. *Secretary.*

### *Local Correspondents*

*Bath* . . Mr. A. D. Bateman, 3 The Circus, Bath.
*Exeter* . . Dr. L. N. Jackson, Mount Jocelyn, Crediton, Devon.
*Gloucester* . Dr. R. F. Jarrett, 9 College Green, Gloucester.
*Plymouth* . Dr. C. R. Croft, Lexden, Hartley Av., Plymouth.
*Taunton* . Dr. M. McAlley, 1 The Crescent, Taunton.
*Torquay* . Dr. A. Robinson Thomas, 20 Devon Square, Newton Abbot, Devon.
*Truro* . . Dr. F. D. M. Hocking, St. Andrews, Perranporth, Cornwall.
*Yeovil* . . Mr. J. A. Vere Nicoll, Knapp House, Yeovil, Somerset.

### NOTICE TO CONTRIBUTORS

Original articles are invited, provided they have not been published elsewhere. Notes of forthcoming events, meetings held, degrees conferred, staff appointments, and other local news are welcomed. The editorial committee reserves the right to make literary corrections.

Illustrations should always be supplied ready for reproduction. All re-touchings or other operations required will be charged to the author. Line drawings should be drawn in Indian ink. We regret that we must ask authors to pay for making blocks, if this is necessary.

J. W. ARROWSMITH LTD., WINTERSTOKE ROAD,
BRISTOL.

### REPORTS FROM SOCIETIES

### WESSEX CLINICAL PATHOLOGISTS

The Wessex branch of the Association of Clinical Pathologists met at Dorchester on 11th May 1963. The President, Dr. T. V. Cooper, had recently designed a new mortuary; this was open for inspection, and the structural plan, materials, and amenities were noted by many as a scheme that might well be followed elsewhere. Dr. A. N. Blades reviewed 12 cases of aplastic anaemia. In 4 cases the aetiology was unknown, but the other 8 were due to toxic substances. The great importance of potentially dangerous drugs was shown by the fact that in 6 cases the toxic substance was a drug, and in 4 of these the drugs were used for the control of psychiatric patients. Dr. G. Tee described the occurrence of cowpox on the face of a boy, whose only contact with cattle was that he had sometimes played around a cowshed.

### BRISTOL PSYCHIATRIC SOCIETY

The Bristol Psychiatric Society was formed by resolution at a meeting held under the chairmanship of Dr. W. Lumsden Walker, at Hortham Hospital on the 11th February 1963. The Constitution states that "The aims of the Society are to promote the discussion of matters of scientific and clinical interest to psychiatrists by arranging meetings and in other ways", and that "All those holding an appointment in psychiatry in the Bristol clinical area shall be regarded as members of the Society". The sole officer of the Society is the Honorary Secretary, who is at present Professor D. Russell Davis, Bristol Royal Infirmary.

At the first meeting Dr. A. G. Baikie (Clinical Effect of Radiation Research Unit, Edinburgh) spoke on "Chromosomes and Mental Subnormality". Cases for discussion were presented by Dr. Grace Woods and others.

### Notes and News

*F.R.C.P.:* Professor D. Russell Davis.
Obituary: A. K. Price, K. H. Pridie, Melvin Roylance, Leonard A. Moore, John Evans.

### Examination Results

*M.R.C.P.:* G. Neale, Julius Smith, A. D. McInnes.

### Appointments

June to December 1962

*UNITED BRISTOL HOSPITALS*

| *Name* | *Appointment* |
|---|---|
| Davis, D. R., M.A., M.D., M.R.C.P., D.P.M. | Professor of Mental Health, Honorary Consultant Psychiatrist. |
| Cornes, J. S., M.D., M.SC., D.C.P. | Lecturer in Pathology and Honorary Consultant Pathologist. |
| Ashworth, B., M.B., CH.B., M.R.C.P. | Senior Registrar in Medicine. |
| Fraser, I. D., M.D., CH.B. | Senior Registrar in Pathology (Haematology). |
| Wills, M. R., M.B., CH.B. | Senior Registrar in Chemical Pathology. |
| Roberts, J. H., M.B., CH.B., D.M.R.D. | Senior Registrar in Radiology. |
| Southwood, W. F. W., M.A., M.B., M.CH., F.R.C.S. | Senior Registrar in Surgery. |
| McInnes, A. D., M.B., CH.B., M.R.C.P.E. | Locum Senior Registrar in Medicine. |
| Arthur, L. J. H., M.B., B.CH., D.O.R.C.O.G., M.R.C.P. | Senior Registrar in Paediatrics. |
| Leslie, Mrs. A., M.B., B.S., D.A., F.F.A.R.C.S. | Registrar in Anaesthetics. |
| Mehta, F. F., B.D.S., L.D.S.R.C.S. | Registrar in Dental Surgery. |
| Peebles, R. A., B.D.S., L.D.S.R.C.S., M.B., B.S. | Registrar in Oral Surgery. |
| Thirkettle, J. L., M.B., B.S., M.R.C.P. | Registrar in Medicine. |
| Harrold, B. P., M.B., B.S. | Registrar in Medicine. |
| Wright, J. G., M.B., CH.B. | Registrar in Psychiatry. |
| Jackson, R. W., M.D. | Registrar in Orthopaedics. |
| Karpouzas, J., M.D. | Registrar in Paediatrics. |
| Tozer, R. A., B.A., M.B., CH.B. | Registrar in Chemical Pathology. |
| Johnson, A. B. C., M.B., CH.B. | Registrar in Radiology. |
| Trapnell, J. E., M.A., N.B., B.CH., F.R.C.S. | Registrar in Surgery. |
| Harris, R. S., M.B., CH.B. | Registrar in Radiology. |
| Barrington, P. C., M.B., B.CHIR. | Locum Registrar in Medicine. |

*SOUTH WESTERN REGIONAL HOSPITAL BOARD*
*BRISTOL*

| | |
|---|---|
| Carter, Stephanie J., M.B., B.S. (Lond.), M.R.C.P., D.M.R.D., F.F.R. | Consultant Radiologist, Cossham-Frenchay Hospital Group. |
| Fairburn, A. C. M.B., B.S., M.R.C.P. (Lond.), D.C.H., D.P.M. | Assistant Psychiatrist (S.H.M.O. grade), Glenside and Barrow Group of Hospitals. |
| Flood, A.R. M.C., M.B., CH.B. (St. Andrews), D.P.M. | Assistant Psychiatrist, (S.H.M.O. grade), Glenside and Barrow Group of Hospitals. |
| Harman, R. R. H. M.B., B.S. (Lond.), M.R.C.P. (Lond.) | Consultant Dermatologist (7 sessions), Bristol Clinical Area and Research Fellow, United Bristol Hospitals—(joint appointment). |

| | |
|---|---|
| Holmes, R. P., M.A., M.B., B.CHIR.(Cantab), D.A., F.F.A.R.C.S. | Consultant Anaesthetist, Cossham-Frenchay Hospital Group. |
| Johnson, D. H., M.D., M.B., CH.B. (Bristol) | Consultant Pathologist, Bristol Clinical Area. |
| Lyons, H. A., M.B., B.CH., B.A.O. (Belfast), M.R.C.P., D.P.M. | Assistant Psychiatrist, (S.H.M.O. grade), Glenside and Barrow Group of Hospitals. |
| O'Connell, N. D., M.B., B.CH. (N.U.I.), D.M.R.D., F.F.R. | Consultant Radiologist, Southmead Hospital Group. |
| Sandry, R. J., M.D., M.B., CH.B. (Bristol) | Consultant Pathologist, Frenchay Hospital. |
| Curry, R., B.A., M.B., B.CHIR., (Camb.), F.R.C.S. (Eng.) | Surgical Registrar, Cossham-Frenchay Hospitals. |
| Draisey, T. F., M.B., CH.B. (Bristol) | Registrar in Pathology, Southmead Hospital. |
| Felton, D. J., M.B., CH.B. (Bristol) | Registrar in Obstetrics and Gynaecology, Southmead Hospital. |
| Forbes, Mehroo, M.R.C.S., L.R.C.P. (Cardiff) | Registrar in Pathology, Frenchay Hospital. |
| Gay, M. J., M.B., CH.B. (Bristol) | Registrar in Child Psychiatry and Mental Sub-normality, Hortham Hospital/Bristol Child Guidance Clinic. |
| Keddie, K. M. G., M.B., CH.B. (Edin.), D.P.M. | Senior Registrar in Psychiatry, Glenside and Barrow Hospitals Group. |
| Rose, M. J., M.B., CH.B. (Bristol), Part I D.P.M. | Registrar in Psychiatry, Glenside and Barrow Hospitals Group. |
| Ryan, Catherine A., M.B., CH.B., B.A.O. (N.U.I.), D.OBST.R.C.O.G., D.A., Primary F.F.A.R.C.S. | Long term locum Registrar in Anaesthetics, Cossham-Frenchay Hospitals. |
| Sumramanian, T., M.B., B.S. (Madras), Primary F.R.C.S. (Edin.) | Surgical Registrar, Weston-super-Mare General Hospital. |

### *NORTH GLOUCESTERSHIRE*

| | |
|---|---|
| Crouch, H. E., M.B., B.CH., B.A.O. (Belfast) | Clinical Assistant in Dermatology (2 sessions), Stroud General Hospital. (Re-appointed). |
| Trickey, S. E., M.A., M.D., M.B., B.CHIR. (Cantab), D.M.R.D., F.F.R. | Consultant Radiologist, North Gloucestershire Clinical Area, based on Cheltenham General Hospital. |

### *BATH*

| | |
|---|---|
| Holman, R. P., M.B., CH.B. (Leeds), M.R.C.P. (Edin.) | Consultant Pathologist, Bath Group of Hospitals. |
| Azzopardi, S. J., M.D. (Malta), D.C.H. (London) | Medical Registrar, Bath Group of Hospitals. |
| Mercer, D., M.B., CH.B. (Sheffield) | Medical Registrar, Bath Group of Hospitals. |
| Morris, H., M.B., CH.B. (Manchester) | Registrar in Psychiatry, Mendip Hospital, Wells. |
| Nixon, Jennifer M., M.B., B.CH.B.A.O. (N.U.I.), D.OBST.R.C.O.G. | Registrar in Obstetrics and Gynaecology, Bath Group of Hospitals. |
| Smith, Barbara G., M.B., CH.B. (Birmingham), D.A. | Anaesthetic Registrar, Bath Group of Hospitals. |
| Spencer, J., M.B., B.S. (Lond.), Primary F.R.C.S. | Surgical Registrar, Bath Group of Hospitals. |
| MacMath, I. F., B.SC. (Glas.), M.B., CH.B., D.OBST.R.C.O.G. (Glas.) | Clinical Assistant in Obst. and Gynae., Trowbridge and Bradford-on-Avon Hospitals. (1 session) re-appointed. |
| Murray, C. W. A., M.B., CH.B. (Edin.), D.C.H., D.OBST.R.C.O.G., D.A. | G.P. Anaesthetist, Trowbridge and District Hospital. |
| Ree, M. J., M.B., CH.B. (Bristol), D.OBST.R.C.O.G., D.C.H. | Clinical Assistant in Paediatrics, Royal United Hospital, Bath (1 session). Re-appointed. |
| Bazeley, R. W., M.B., B.S. (Lond.)<br>Burrowes, W. L., M.D., B.CH., M.R.C.P.<br>Pinching, J., B.M., B.CH., M.R.C.P.<br>Ronn, H. H., M.B., B.S. (Lond.), M.R.C.P. | all Clinical Assistants in General Medicine, Bath. Appointed for one year. (1 session each). |

### SOUTH SOMERSET

| | |
|---|---|
| Nicholson, R. D., M.B., B.S. (Lond.), F.R.C.S. (Eng.) | Clinical Assistant in Surgery, Minehead and West Somerset Hospital. (3 sessions). |
| Conway, Stella M., L.R.C.P., L.R.C.S. (Edin.), L.R.F.P.S. (Glasgow), D.P.M. | Senior Registrar in Psychiatry, Tone Vale Hospital. |
| Flynn, D. M., M.B., CH.B. (Edin.), D.C.H. (Glasgow) | Registrar in Paediatrics, Taunton and Somerset Hospital (joint appt. with The London Hospital). |
| Smith, G. O., M.B., B.S. (Melbourne), M.R.C.O.G. | Registrar in Obstetrics and Gynaecology, Taunton and Somerset Hospital. |

### DEVON AND EXETER

| | |
|---|---|
| Crow, R. S., M.D., CH.B. (Aberdeen), M.R.C.P. (London) | Consultant General Physician, Torbay Hospital, Torquay. |
| Lewars, P. H. D., M.B., B.S., F.D.S., R.C.S. (Eng.) | Consultant Dental Surgeon, Devon and Exeter Clinical Area. |
| Bond, A., M.B., CH.B. (Leeds), D.OBST.R.C.O.G., Primary F.F.A.R.C.S. | Registrar in Anaesthetics, Royal Devon and Exeter Hospital. |
| Byles, A. B., B.SC., M.B., B.S. (Lond.), D.OBST.R.C.O.G. | Registrar in Obstetrics and Gynaecology, Royal Devon and Exeter Hospital/Southmead Hospital (rotating appt.). |
| Dubourg, G. O., M.B., CH.B. (Liverpool), D.P.M. | Senior Registrar in Psychiatry, Exminster Hospital. |
| Harse, H. R., M.D. (Alberta), L.M.C.C. (Canada). | Surgical Registrar, Torbay Hospital, Torquay. |
| Hepple, F. J. B., M.B., B.S. (Queensland), Primary F.R.C.S.E. | Surgical Registrar, Royal Devon and Exeter Hospital. |
| Hill, D. J., M.B., B.S. (Lond.), D.A. | Anaesthetic Registrar, Torbay Hospital. |
| Piggot, T. A., M.B., B.CH., B.A.O. (Belfast), F.R.C.S.E. | Registrar in Orthopaedic and Traumatic Surgery, Torbay Hospital/Princess Elizabeth Orthopaedic Hospital, Exeter (rotating appointment). |
| Townsend, A. C., M.A., M.B., B.CH. (Cantab), F.R.C.S. (Eng.) | Senior Orthopaedic Registrar, Princess Elizabeth Orthopaedic Hospital, Exeter. |
| Vickery, C. M., M.B., B.S. (Lond.), F.R.C.S. (Eng.) | Senior Surgical Registrar, Royal Devon and Exeter Hospital/Southmead Hospital/ Teaching Hospital (joint appointment with the United Bristol Hospitals). |

### PLYMOUTH

| | |
|---|---|
| Dawson, D. A., M.B., B.S. (Lond.), F.R.C.S. (Eng.) | Consultant E.N.T. Surgeon Plymouth Clinical Area (also covering the Torquay area). |
| Hadley, K. J., B.SC., M.B., CH.B. (Birmingham), F.R.C.S. (Eng.) | Consultant E.N.T. Surgeon, Plymouth Clinical Area (and covering the Launceston area). |
| Keddie, F. S., M.A., B.M., B.CH. (Oxon), F.F.A.R.C.S. | Consultant Anaesthetist, Plymouth Clinical Area. |
| Neville, R., M.R.C.S., L.R.C.P., D.P.M. (Conjoint Board). | Assistant Psychiatrist (S.H.M.O. grade), Moorhaven Hospital, Ivybridge. |
| Reece, M. W., M.B., B.S. (Lond.), F.R.C.S | Consultant Surgeon, Plymouth Clinical Area. |
| Weeks, K., M.B., B.S., D.P.M. (Lond.) | Consultant Psychiatrist, Plymouth Mental Health Centre. |
| Bethell, M. S., M.B., B.CHIR., (Cantab), D.P.M. | Senior Registrar in Psychiatry, Moorhaven Hospital, Ivybridge. |
| Rasaretnam, R., M.B., B.S. (Lond.), F.R.C.S. (Edin.) | Surgical Registrar, Freedom Fields Hospital. |
| Smith, D. H., M.B., B.S. (Lond.) | Registrar in General Medicine, and Infectious Diseases, South Devon and East Cornwall Hospital/Scott Hospital. |
| Voyce, M. A., B.SC. (Bristol), M.B., CH.B. (Bristol) | Registrar in Paediatrics, Freedom Fields Hospital. |

### *WEST CORNWALL*

| | |
|---|---|
| Dessart, J. E., M.B., B.S. (Lond.), D.P.M. (England) | Consultant Psychiatrist in Child Guidance, West Cornwall Clinical Area. |
| Rowe, L. F. W., M.B., CH.B. (Edin.), D.P.M. (Conjoint Board) | Consultant Psychiatrist, St. Lawrence's Hospital, Bodmin. |
| Ahmad, B., M.B., B.S. (Punjab), D.A. (Copenhagen), D.A. (Lond.) | Anaesthetic Registrar, Royal Cornwall Infirmary. |
| Hassanein, M. R., M.B., B.CH. (Cairo), Primary F.R.C.S.E. | Registrar in Orthopaedics, Royal Cornwall. Infirmary. |
| Maddox, C. McL., M.B., B.CHIR. (Cantab), Primary F.R.C.S. | Surgical Registrar, Royal Cornwall Infirmary. |
| Uzodike, B. C., M.B., CH.B. (Leeds), D.OBST.R.C.O.G. | Registrar in Obstetrics and Gynaecology, Camborne-Redruth Hospital. |

### *NORTH GLOUCESTERSHIRE*

| | |
|---|---|
| Bannerjee, D. B., B.SC., M.B., B.S. (Lucknow), D.C.H. | Medical Registrar, Cheltenham General Hospital. |
| Hocking, Elizabeth D., M.A., M.B., B.CHIR. (Cantab) | Medical Registrar, Gloucestershire Royal Hospital. |
| Islam, A. K. M. S., M.B., B.S. (Calcutta) | Registrar in Geriatrics and General Medicine, Gloucestershire. |

Printed by

J. W. Arrowsmith Ltd., Bristol

